# Clinical experience across the fetal‐fraction spectrum of a non‐invasive prenatal screening approach with low test‐failure rate

**DOI:** 10.1002/uog.21904

**Published:** 2020-09-01

**Authors:** S. Hancock, R. Ben‐Shachar, C. Adusei, C. B. Oyolu, E. A. Evans, H. P. Kang, C. Haverty, D. Muzzey

**Affiliations:** ^1^ Myriad Women's Health South San Francisco CA USA; ^2^ Myriad Genetics Salt Lake City UT USA

**Keywords:** cell‐free DNA, fetal fraction, non‐invasive prenatal screening, outcome collection, whole‐genome sequencing

## Abstract

**Objective:**

To describe our clinical experience across the entire range of fetal‐fraction (FF) measurements of a non‐invasive prenatal screen (NIPS) that uses whole‐ genome sequencing (WGS).

**Methods:**

We analyzed retrospectively results from 58 105 singleton pregnancies that underwent NIPS on a customized WGS platform during an 8‐month period and assessed clinical test performance for trisomy 21, trisomy 18 and trisomy 13. Pregnancy outcomes were sought for all screen‐positive patients and for 18% of screen‐negative patients. As differences in outcome‐collection response rates could artificially impact test‐performance calculations, we computed inferred sensitivity, specificity, positive predictive values (PPV) and negative predictive values adjusted for ascertainment bias.

**Results:**

The screening test yielded a result for 99.9% (*n* = 58 048) of patients, meaning that approximately 1 in 1000 patients received a test failure (i.e. test failure rate = 0.1%). Of pregnancies with a test result, 572 (1%) screened positive for one of the common aneuploidies (362 for trisomy 21, 142 for trisomy 18 and 68 for trisomy 13). Informative outcomes were received for 237 (41.4%) patients with a screen‐positive result and 3258 (5.7%) of those with a screen‐negative result. In the full cohort, inferred sensitivities for trisomy 21, trisomy 18 and trisomy 13 were 99.7%, 96.8% and 94.3%, respectively, and PPVs were 93.1%, 85.2% and 48.4%, respectively. If a FF threshold of 4% had been employed to guard against false negatives, calculated sensitivities for the three aneuploidies would not have changed significantly, yet, importantly, the overall test‐failure rate would have increased to 6.6% (*n* = 3829), impacting 1 in 15 women.

**Conclusions:**

Our clinical experience demonstrates that a customized WGS‐based NIPS without a FF threshold achieves high accuracy while maintaining a low test‐failure rate of 0.1%. As such, alternative strategies to ensure high accuracy of detection of common aneuploidies in samples with low FF (such as redraw after test failure, redrawing at a later gestational age, risk scoring based on FF) are not necessary for this screening approach. © 2019 The Authors. *Ultrasound in Obstetrics & Gynecology* published by John Wiley & Sons Ltd on behalf of the International Society of Ultrasound in Obstetrics and Gynecology.

## Abstract 2

Linked article: There is a comment on this article by Jelsema *et al.* Click here to view the Correspondence.

**CONTRIBUTION**

*What are the novel findings of this work?*

This study directly explores pregnancy outcomes following non‐invasive prenatal screening (NIPS) of samples with low fetal fraction (FF). On most NIPS offerings, low‐FF samples are failed, yet we found that a customized whole‐genome sequencing (WGS)‐based NIPS that does not fail samples based on FF has comparable sensitivity and specificity for high‐ and low‐FF samples.

*What are the clinical implications of this work?*

NIPS test failures due to low FF increase patient anxiety, lengthen the prenatal screening process and complicate provider workflow. Our study shows that a customized WGS‐based NIPS approach that does not utilize a FF threshold performs comparably at high and low FF, rendering test failures due to low FF unnecessary.

## INTRODUCTION

Non‐invasive prenatal screening (NIPS) based on cell‐free DNA (cfDNA) is rapidly replacing traditional methods of aneuploidy screening in clinical practice^1^, ^2^. Many laboratories offer NIPS using different molecular methods^3^, ^4^, ^5^, and clinical‐experience studies consistently demonstrate high accuracy and positive predictive value (PPV) for aneuploidies involving chromosomes 21, 18 and 13^6^, ^7^, ^8^. However, these studies share common shortcomings, such as lack of active follow‐up on screen‐negative results, which could overestimate the sensitivity of the method by assuming euploidy, as well as limited consideration for the implications of test failure^6^, ^7^, ^8^.

NIPS test failures (or ‘no calls’) are often attributed to an insufficient fraction of pregnancy‐derived genomic material in the cfDNA (‘fetal fraction’ (FF))^9^. A low FF (< 4%, as classified by the American College of Medical Genetics and Genomics^10^) can prompt a test failure due to reduced aneuploidy sensitivity, but the extent of sensitivity drop is platform‐dependent^11^. Many laboratories establish a low‐FF threshold below which all samples are failed; reported thresholds range from 2.8% to 4.0% FF and yield failure rates of between 1.9% and 5.2%^8^, ^12^. Test failures are clinically problematic because they require additional follow‐up and delay the identification of at‐risk pregnancies. Currently, guidelines recommend that providers should offer patients with NIPS test failure the same invasive follow‐up that is offered to patients with a screen‐positive result (i.e. chorionic villus sampling (CVS) or amniocentesis)^10^, ^13^. Consequently, a NIPS with an elevated failure rate negates one of the chief benefits of non‐invasive testing, which is a reduction in invasive procedures and associated pregnancy loss^14^, ^15^, ^16^. Additionally, patients who do not pursue invasive follow‐up may lose the opportunity to make informed pregnancy‐management decisions if they receive either delayed results after sample redraw(s) or no results at all^13^.

The reported failure rate for NIPS using whole‐genome sequencing (WGS) is as low as 0.1%^6^, ^17^, but clinical test performance at low FF is unexplored. Such evaluation must be laboratory‐specific because WGS‐based platforms are not interchangeable since they use different next‐generation sequencing protocols, data‐analysis algorithms and data review to provide patient results^3^, ^18^. Here, we present our clinical experience with a customized WGS‐based NIPS method that does not utilize a FF threshold and has a low test‐failure rate for both screen‐positive and screen‐negative patients.

## METHODS

### Dataset

We retrospectively analyzed anonymized data from 58 105 patients who underwent NIPS over an 8‐month period from 1 June 2017 to 31 January 2018. We utilized a WGS‐based approach (adapted from Fan *et al*.^19^), in which the relative abundance of cfDNA in maternal plasma was measured and *Z*‐score was used to quantify any significant chromosome‐specific deviations in cfDNA abundance consistent with fetal aneuploidy. Chromosome analysis results for trisomies were reported as ‘screen‐positive’ or ‘screen‐negative’. FF was measured in each sample using an adaptation of a previously described regression‐based approach^20^, trained on our laboratory's data. A FF threshold was not employed as a reason for test failure; however, test failure could occur for a variety of technical reasons, such as genomic DNA occluding cfDNA signal, poor sequencing quality and low number of mapped sequencing reads.

Twin pregnancies (*n* = 1257) and patients with a screen‐positive result for multiple aneuploidies (*n* = 8) were excluded from the dataset. However, pregnancies with a confirmed or suspected vanishing twin and/or mosaicism were included. All patients provided informed consent for testing and anonymized research. All samples from New York State were excluded from this analysis. This study was exempt from institutional review board (IRB) oversight (reviewed and designated by Western IRB).

### Outcome collection

Pregnancy outcomes were obtained via our laboratory's routine Health Insurance Portability and Accountability Act (HIPAA)‐compliant process for continuous quality improvement and best practices (including the logging of self‐reported false negatives (FNs)), as well as through targeted efforts for the purpose of this study. Forms requesting pregnancy outcome information were automatically faxed to ordering offices and requested the following information: whether invasive diagnostic testing was performed (e.g. CVS and/or amniocentesis), and if so, the results; the outcome of the pregnancy (e.g. ongoing, spontaneous miscarriage, intrauterine fetal demise, stillbirth, elective termination of pregnancy, preterm delivery, full‐term delivery); the sex of the fetus/child (via ultrasound, newborn examination and/or chromosome analysis); and any additional information (Figure S1). Medical records could be returned with the outcome‐collection form.

We actively sought pregnancy outcome information for all pregnancies with a screen‐positive result for the common aneuploidies (trisomy 21, trisomy 18, trisomy 13) and for a subset of randomly selected pregnancies with a screen‐negative result. The outcome‐request volume for screen‐negative pregnancies was capped to two per week on a per‐clinic basis, to limit workflow burden, but it was otherwise random (18% were requested in total). Initial requests were faxed 3 weeks after screen‐positive results were released and 30 weeks after screen‐negative results were released. If a response was not received within 2 weeks of the initial fax send date, a follow‐up request form was sent for a total of two automatic attempts. Manual faxes and/or phone calls by certified genetic counselors were also performed. Outcome responses obtained were then recorded in a HIPAA‐compliant internal database and anonymized for the purposes of the study.

### Concordance evaluation

Two certified genetic counselors, experienced in prenatal genetics, independently reviewed the concordance/discordance between screening results and pregnancy outcomes. The screening result was classified as ‘concordant’ if it was consistent, or ‘discordant’ if it was inconsistent, with the prenatal or postnatal diagnostic chromosome analysis or newborn examination. Cohen's kappa statistic was calculated to measure inter‐rater reliability of the evaluation of concordance. For cases without diagnostic chromosome analysis available, screen‐negative results were additionally classified as ‘concordant’ if the provider reported a pregnancy outcome of ‘term delivery’ or ‘preterm birth’ with no signs of trisomy 21, 18 or 13 based on newborn examination.

### Analysis of test performance

Measures of test performance (sensitivity, specificity, PPV and negative predictive value (NPV)) depend on the relative number of true positives (TPs), true negatives (TNs), false positives (FPs) and FNs. Differences in outcome‐collection response rates among screen‐positive patients, screen‐negative patients and self‐reported FNs could artificially impact on test‐performance calculations (for example, if all FN outcomes are reported but only half of TP outcomes are reported, then the sensitivity would be underestimated). Therefore, the numbers of FNs and FPs were adjusted to calculate inferred sensitivity, inferred specificity, inferred PPV and inferred NPV, using a similar methodology to that described by Taneja *et al*.^6^. Details of the adjustment methodology used are provided in Appendix S1. In brief, to calculate inferred sensitivity (TP/(TP + FN_sensitivity‐adjusted_)), the number of FNs was adjusted based on the rate of informative outcomes received for screen‐positive patients (e.g. if 50% of screen‐positive patients reported outcomes and 100% of FNs were reported, then we scaled down the number of FNs by 50% to yield FN_sensitivity‐adjusted_). To calculate inferred specificity (TN/(TN + FP_specificity‐adjusted_)), the number of FPs was adjusted based on the relative rate of informative outcomes received for screen‐negative patients compared with that for screen‐positive patients. To calculate inferred NPV (TN/(TN + FN_NPV‐adjusted_)), the number of FNs was adjusted based on the rate of informative outcomes received for screen‐negative patients. No adjustment was needed for the PPV calculation (TP/(TP + FP)) because both TP and FP outcomes were assumed to be received at the same rate. We evaluated directly the impact of our response‐rate assumptions and associated values of the adjustments for FN and FP (Table S1), and found that, in general, our assumptions had negligible impact on the conclusions of the study; for instance, if our assumption of 100% FN reporting was grossly incorrect and only 60% of FNs were reported, the inferred sensitivity would change by < 1%.

We implemented several strategies to assess confidence in our calculated performance metrics. For both inferred sensitivity and inferred specificity, to account for the impact of incomplete outcome‐data collection, we calculated uncertainty ranges by assuming that unknown outcomes (e.g. from patients whose providers did not return an outcome form) for screen‐positive results were discordant (lower bound) or concordant (higher bound). Additionally, because inferred sensitivity and inferred specificity metrics calculated at low FF considered only a subset of the data and accordingly involved relatively few positive samples, we determined CIs by repeated sampling from the data based on the frequency of observed positives (Appendix S1 and Table S2). As an example, this bootstrapping simulation analysis revealed that our data at low FF were sufficient to yield a 95% CI for trisomy 21 inferred sensitivity of approximately ± 7%. For PPV and inferred NPV, 95% CIs were computed assuming that the measurements were normally distributed (standard normal interval).

A simulated power analysis was performed to determine the conditions under which differences in inferred sensitivity between patients with FF < 4% and those with FF ≥ 4% would be expected to yield a significant difference (Appendix S1). Differences in test performance of subpopulations (FF < 4% and FF ≥ 4%) were computed using either the proportions *Z*‐test (Statsmodels version 0.10.1) or Fisher's exact test (Scipy version 1.3.0). Differences in inferred sensitivity and PPV were computed using Fisher's exact test. Differences in inferred specificity and inferred NPV were computed using proportions *Z*‐test. We calculated the ratio of the FN rate (FN/(FN + TP)) in patients with FF < 4% to the FN rate in patients with FF ≥ 4%, as well as the corresponding 95% CIs (Appendix S1). All analysis was performed using Python version 2.7.10, Numpy version 1.13.1 and Pandas version 0.20.3.

## RESULTS

We analyzed retrospectively samples from 58 105 patients who underwent NIPS during the study period. Our customized WGS‐based NIPS yielded a result for 58 048 patients (99.9%). The remaining 57 patients had a test failure due to technical reasons, thus the test failure rate was 0.1%. Of the patients with a result, 572 (1%) cases screened positive for one of the common aneuploidies: 362 for trisomy 21, 142 for trisomy 18 and 68 for trisomy 13 (Table 1). Median turnaround‐time was 4 calendar days (interquartile range, 3.2–4.8 days (95% CI, 2.3–8.1) days).

**Table 1:** Non‐invasive prenatal screening (NIPS) results at time of patient screening and comparison with pregnancy outcome in 58 105 patients who underwent a customized whole‐genome sequencing‐based NIPS without a fetal‐fraction (FF) threshold

NIPS result	Trisomy 21	Trisomy 18	Trisomy 13	Total
Screen positive	362	142	68	572
Outcome obtained	153 (42)	53 (37)	31 (46)	237 (41)
True positive	146	45	15	206
FF < 4%	10	7	2	19
False positive	7	8	16	31
FF < 4%	1	2	3	6
Screen negative	57 686	57 906	57 980	57 476
Outcome obtained	3342 (6)	3442 (6)	3464 (6)	3258 (6)
True negative	3339	3437	3462	3248
FF < 4%	245	248	254	229
False negative	3	5	2	10
FF < 4%	3	2	0	5
Test failure	—	—	—	57

Data are given as *n* or *n* (%).

**Figure 1:**
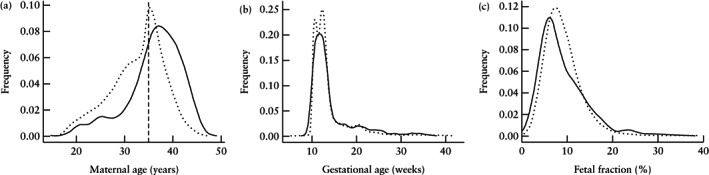
Distribution of maternal age (a), gestational age (b) and fetal fraction (c) in 58 105 patients who underwent customized whole‐genome sequencing‐based non‐invasive prenatal screening, according to whether they were screen positive (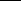) or screen negative (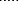) for trisomy 21, 18 or 13. Traces indicate Gaussian kernel‐smoothed data for clarity. Vertical dashed line in (a) indicates advanced maternal‐age threshold of 35 years.

Figure 1 shows the distribution of maternal age, gestational age and FF for screen‐positive and screen‐negative patients. Screen‐positive patients were significantly older and had significantly lower FF than did screen‐negative patients (*P* < 0.0001 and *P* < 0.0001, respectively; one‐sided Kolmogorov–Smirnov test). There was no significant difference in gestational age at the time of screening between patients with a positive and those with a negative result (*P* = 0.1; Kolmogorov–Smirnov test). Maternal age ranged between 18 years and 45 years in our cohort, and gestational age ranged between 10.0 weeks and 41.5 weeks. FF was in the minimum reportable FF range of our assay (< 1%) in 107 patients and in the maximum reportable FF of our assay (> 30%) in 85 patients.

### Outcome collection

To assess the accuracy of screen‐positive and screen‐negative reports, pregnancy outcomes were requested from all cases with a screen‐positive result and from 18% of those with a screen‐negative result. Of these, informative outcomes were received for 237 screen‐positive patients and 3258 screen‐negative patients (41% and 30% of those requested, respectively).

Table 1 summarizes our clinical experience in terms of TP, TN, FP and FN based on the results reported to the patients at the time of screening. In total, 3248 TNs, 206 TPs, 10 FNs and 31 FPs were observed. Two TPs, two FPs and one FN were pregnancies with a vanishing twin and/or mosaicism. Outcome classification as TP, TN, FP or FN was concordant in all cases but one, which was adjudicated by a third certified genetic counselor. Cohen's kappa statistic was 0.981 for total cohort concordance.

All observed FNs in the patient cohort were proactively reported by the provider; no additional FNs were discovered among the screen‐negative patients for whom outcomes were requested. The distributions of maternal age, gestational age and FF were very similar between patients with and those without reported outcomes (Figure S2), suggesting that performance metrics inferred from the reported outcomes reflect those of the whole cohort.

**Figure 2:**
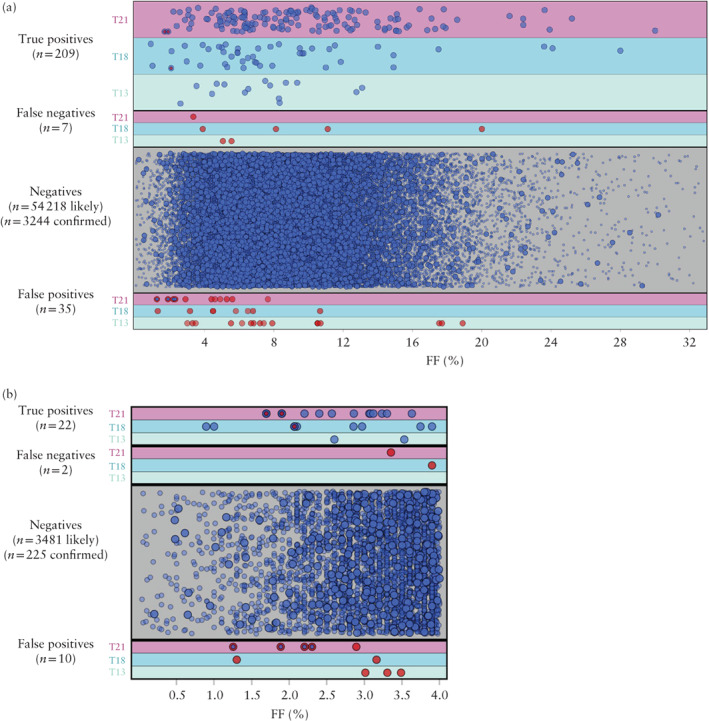
Performance of customized whole‐genome sequencing‐based non‐invasive prenatal screening method without fetal‐fraction (FF) threshold, based on reanalysis of patient samples using updated algorithm, for whole patient cohort (a) and for patients with FF < 4% (b). Blue circles represent samples with probable (small circles) or confirmed (large circles) concordant result with pregnancy outcome and red circles represent samples for which result was discordant compared with pregnancy outcome. Samples for which test result changed based on updated algorithm, compared with result at time of patient screening, are indicated by blue/red smaller circles inside red/blue larger circle. Specifically, in three samples represented by large blue circle with small red circle, result was screen negative at time of patient screening (false negative as pregnancy outcome was positive for aneuploidy) and became true positive after reanalysis. In four samples represented by large red circle with small blue circle, result was correctly identified as screen negative at time of patient screening and became false positive after reanalysis using the updated algorithm. In (a), measurements are not shown for 51 patients with FF > 32% and 355 screen‐positive patients for whom no informative outcomes were obtained. In (b), out of 64 screen‐positive patients with FF < 4%, results are not shown for 32 because no informative outcomes were obtained. A random vertical offset is provided for visualization purposes and has no clinical meaning. T13, trisomy 13; T18, trisomy 18; T21, trisomy 21.

Algorithm improvements are routinely integrated into the NIPS calling methodology, and one set of improvements was deployed during the study period. Table 2 shows performance data for the entire patient cohort when samples were reanalyzed using the updated algorithm. Using the updated algorithm, three low‐FF (FF < 4%) FNs would have been correctly identified as screen positive, and four low‐FF TNs would have been FPs with a screen‐positive result. Figure 2a displays the FF values of confirmed TNs (*n* = 3244), TPs (*n* = 209), FNs (*n* = 7) and FPs (*n* = 35) for the three common trisomies, based on the results of the updated algorithm. The distributions of FF for TNs and TPs mirrors that in the full cohort shown in Figure 1, with the FF of TPs ranging from 0.9% to 30.0%. Consistent with reports of a range of biological factors (e.g. mosaicism) that can cause false NIPS results at any FF level^21^, ^22^, the FF of FNs ranged from 3.4% to 20.0% and the FF of FPs ranged from 1.2% to 18.9% (Figure 2a). Because our calling algorithm does not fail samples because of low FF alone, we closely scrutinized the test performance specifically for those samples with FF < 4% using the updated algorithm. Of these, there were 225 TNs, 22 TPs, two FNs and 10 FPs (Figure 2b, Table 2).

**Table 2:** Non‐invasive prenatal screening (NIPS) results using updated whole‐genome sequencing‐based NIPS algorithm and comparison with pregnancy outcome in 58 105 patients who underwent a customized whole‐genome sequencing‐based NIPS without a fetal‐fraction (FF) threshold

NIPS result	Trisomy 21	Trisomy 18	Trisomy 13	Total
Screen‐positive	368	143	68	579
Outcome obtained	159 (43)	54 (38)	31 (46)	244 (42)
True positive	148	46	15	209
FF < 4%	12	8	2	22
False positive	11	8	16	35
FF < 4%	5	2	3	10
Screen‐negative	57 680	57 905	57 980	57 469
Outcome obtained	3336 (6)	3341 (6)	3464 (6)	3251 (6)
True negative	3335	3437	3462	3244
FF < 4%	241	248	254	225
False negative	1	4	2	7
FF < 4%	1	1	0	2
Test failure	—	—	—	57

Data are given as *n* or *n* (%).

### Inferred clinical test performance

We next analyzed inferred clinical test performance using the updated algorithm. Inferred sensitivities for the full cohort were highest for trisomy 21 (99.7%), followed by trisomy 18 (96.8%) and trisomy 13 (94.3%) (Table 3). Inferred specificity exceeded 99.9% for all aneuploidies (Table 3). PPV for trisomy 21, trisomy 18 and trisomy 13 were 93.1%, 85.2% and 48.4%, respectively; inferred NPV for all common aneuploidies exceeded 99.98%. Corresponding inferred clinical test performance based on the original algorithm used at the time of patient testing is shown in Table S3.

**Table 3:** Inferred test performance of updated whole‐genome sequencing‐based non‐invasive prenatal screening algorithm

	Aneuploidy			
	Trisomy 21	Trisomy 18	Trisomy 13	Total
Inferred sensitivity				
All patients	99.7 (99.3–99.7)	96.8 (92.0–97.1)	94.3 (88.2–96.3)	98.6 (96.8–98.7)
Patients with FF < 4%	95.5 (92.3–96.2)	94.8 (88.9–95.5)	100 (n/a)*	95.7 (91.7–99.2)
Patients with FF ≥ 4%	100 (100–100)	97.2 (92.7–97.4)	93.4 (86.7–95.7)	98.9 (97.4–99.0)
Inferred specificity				
All patients	99.96 (99.62–99.98)	99.96 (99.83–99.99)	99.94 (99.91–99.97)	99.86 (98.36–99.94)
Patients with FF < 4%	99.76 (99.52–99.87)	99.88 (99.60–99.95)	99.82 (98.76–99.92)	99.46 (98.87–99.73)
Patients with FF ≥ 4%	99.97 (99.63–99.99)	99.97 (99.85–99.99)	99.95 (99.92–99.98)	99.89 (99.39–99.95)
PPV				
All patients	93.1 (89.1–97.0)	85.2 (75.7–94.7)	48.4 (30.8–66.0)	85.7 (81.3–90.1)
Patients with FF < 4%	70.6 (48.9–92.2)	80.0 (55.2–100)	40.0 (0.0–82.9)	68.8 (52.7–84.8)
Patients with FF ≥ 4%	95.8 (92.5–99.1)	86.4 (76.2–96.5)	50.0 (30.8–69.2)	88.2 (83.9–92.5)
Inferred NPV				
All patients	> 99.99 (99.98–100)	99.99 (99.97–100)	> 99.99 (99.98–100)	99.99 (99.95–100)
Patients with FF < 4%	99.97 (99.77–100)	99.97 (99.77–100)	100 (n/a)*	99.95 (99.64–100)
Patients with FF ≥ 4%	100 (n/a)*	99.99 (99.97–100)	> 99.99 (99.98–100)	99.99 (99.96–100)

Data are % (uncertainty range) for inferred sensitivity and inferred specificity, and % (95% CI) for inferred PPV and inferred NPV.

* Uncertainty range or CI not shown owing to small sample size.

Inferred test performance is adjusted for ascertainment bias, as described in Methods and Appendix S1. n/a, not applicable; NPV, negative predictive value; PPV, positive predictive value.

To assess test performance at low FF, we compared the respective results for patients with FF < 4% and those with FF ≥ 4%. As shown in Table 3, the average performance metrics were comparable for FF < 4% and FF ≥ 4%, and most uncertainty ranges overlapped. However, to determine if our comparisons of sensitivity and specificity for FF < 4% and FF ≥ 4% were meaningful, we pursued two strategies to directly address the presence of relatively few aneuploidies among low‐FF samples (especially for trisomy 13, for which there were two TPs and no FNs; Table 2).

First, we calculated the CIs (distinct from the uncertainty ranges shown in Table 3) of inferred sensitivity and inferred specificity for low‐FF samples and observed that they were comparable with the uncertainty ranges reported in Table 3 (Table S2). Importantly, the lower 95% CI boundaries of inferred sensitivity for trisomies 18 and 21 for low‐FF samples were 84.0% and 86.2%, respectively (Table S2), suggesting that there was sufficient sampling of these aneuploidies at low FF to reveal confidently high sensitivity (there were not enough aneuploid samples for trisomy 13 to calculate CI).

Second, based on the size of our cohort, we determined the power of our study to detect a statistically significant and clinically meaningful difference in inferred sensitivity for FF < 4% and for FF ≥ 4%. This analysis was important because a poorly powered analysis could, for example, expect to detect a significant difference only if sensitivity were, say, 100% at high FF and 25% at low FF, thereby rendering effectively meaningless an observation of no significant change. We performed this analysis on the three trisomies combined and found that we had sufficient power (20% beta and 5% alpha; Appendix S1) to expect significantly different results between FF < 4% and FF ≥ 4% if the low‐FF sensitivity were ≤ 81.7%. Put differently, for low‐FF sensitivity values in excess of 81.7%, we would expect to see no significant difference in sensitivity for FF < 4% and FF ≥ 4%. Indeed, we observed a sensitivity of 95.7% in low‐FF samples, and this difference was not significantly different from the sensitivity in high‐FF samples (*P* > 0.29; Fisher's exact test). We further determined that the 95% CIs of the ratio of FN rates at low FF and high FF were not significantly different (Appendix S1). Taken together, these results demonstrate that, in our cohort of over 58 000 patients, the sensitivity for common aneuploidies is not significantly different above or below a FF of 4%.

For all aneuploidies combined and for trisomy 21 alone, we did not observe a significant difference in inferred specificity or inferred NPV (*P* > 0.1) above and below a FF of 4%. In both the FF < 4% and FF ≥ 4% groups, PPV for trisomy 21 was > 70% and overall PPV was > 68%; however, the values between FF < 4% and FF ≥ 4% groups were significantly different (Fisher's exact test, *P* < 0.01 and *P* < 0.05, respectively).

### Consequences of FF threshold

**Figure 3:**
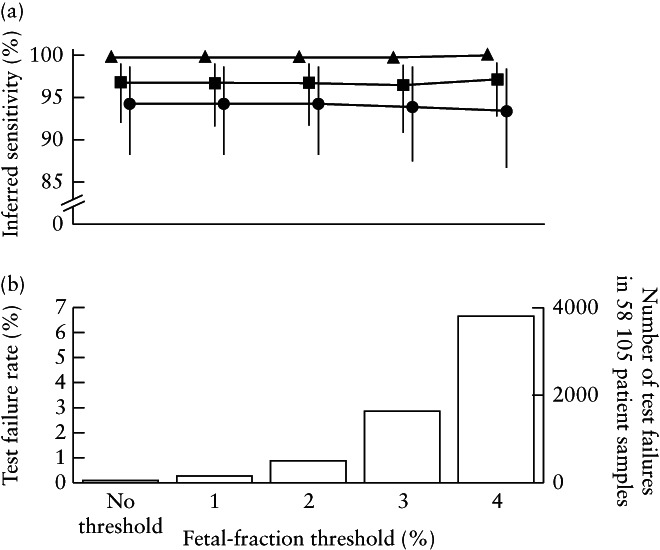
Clinical sensitivity for trisomy 21 (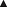), trisomy 18 (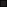) and trisomy 13 (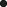) (a) and rate and number of test failures that would occur (b) if fetal‐fraction threshold of 0%, 1%, 2%, 3% or 4% was utilized in whole‐genome sequencing‐based non‐invasive prenatal screening method. Data are for 58 105 patient samples reanalyzed using updated algorithm.

To determine if a FF threshold would improve the test performance of our assay, we calculated the inferred sensitivities and the number of test failures that would have occurred if a FF threshold of 0%, 1%, 2%, 3% or 4% was utilized (Figure 3). The highest FF threshold of 4% would have yielded no significant change in inferred sensitivity or inferred specificity for each individual aneuploidy. Inferred sensitivity for trisomy 21 and trisomy 18 would increase non‐significantly by 0.3% and 0.4%, respectively (proportion *Z*‐test, *P* > 0.5), and observed sensitivity for trisomy 13 would decrease by 0.9% (proportion *Z*‐test, *P* = 0.9) because no FNs for trisomy 13 were observed for patients with FF < 4%. Critically, a 4% FF threshold would lead to 3829 total test failures using our assay, giving an overall test failure rate of 6.6% compared with 0.1% when no FF threshold is used (Figure 3). In sum, we show that a FF threshold > 0% would increase the percentage of NIPS test failures (up to 65‐fold for a 4% threshold) while not significantly altering sensitivity.

## DISCUSSION

While many clinical‐experience studies have demonstrated the high accuracy of NIPS, none has thoroughly investigated the performance of NIPS at low FF. Such an investigation is not possible for many NIPS offerings because all samples below a FF threshold receive a test failure result. In this study of over 58 000 patients, we examined the clinical performance of a WGS‐based NIPS customized to be sensitive and specific across the entire empirical FF range, such that a low FF threshold was not required. In addition to spanning the range of FF values, the diverse, unbiased patient cohort analyzed here included a wide spectrum of maternal and gestational ages, without excluding pregnancies with mosaicism or a twin demise. While these results may not be generalizable to other NIPS laboratories, it is important to note that we have shown not only that the general clinical performance of this screening test is consistent with other characterizations of NIPS^6^, ^8^, ^9^, but also that inferred sensitivity and inferred specificity are not significantly different between patients with a FF < 4% and those with a FF ≥ 4%.

It is common for cohort studies to provide summary statistics of the patient population (e.g. average, spread and extrema of maternal age), but uncommon to show the metrics' full distributions, which illustrate features of the data that summary statistics do not convey. Because positive results are rare in a screening test, it is crucial to highlight the distribution of screen‐positive patients. If patients with a screen‐positive result have a different gestational age, risk profile (e.g. abnormal ultrasound) or FF from screen‐negative patients, as was the case in a recent study evaluating NIPS performance for the detection of 22q11.2 deletion syndrome^23^, then calculations of sensitivity, PPV and other test performance metrics may not reflect the true values in a general population. In the current study, screen‐positive patients did not have significantly different gestational ages compared with screen‐negative patients, but they did have a significantly lower FF distribution, as expected, owing to a reduced FF in trisomies 13 and 18^24^. Thus, calculated performance metrics from our cohort are expected to be representative of general population screening.

Although complete collection of outcomes was limited by the availability of certain information (e.g. patients lost to follow‐up, possible discordant results from fetal demise without chromosome analysis, etc.) and by 30% provider response to requests for outcomes on negative results, our outcome‐collection process had a broader scope than did several previous studies^6^, ^7^, ^8^. We actively sought follow‐up for 18% of all screen‐negative samples and found that providers were cooperative in this effort, enabling us to directly evaluate the assumption in past clinical‐experience studies that all FN reports would be voluntarily reported^6^. Our experience upholds this assumption, as no FNs beyond those that were self‐reported were captured in the 3248 (0% (95% CI, 0.00–0.08%); Jeffreys interval) screen‐negative outcomes received by our laboratory.

The molecular and computational approaches employed by NIPS laboratories can differ^12^, ^18^, ^25^, even among those utilizing WGS‐based NIPS, and these differences can cause discrepancies in test performance^6^, ^7^, ^8^. Importantly, each laboratory also has the option of trade‐off between sensitivity, specificity and test‐failure rate (e.g. calling every sample screen‐positive would result in 100% sensitivity and 0% test failures, but at the cost of achieving a specificity of only ∼ 1%). Yet, there is no agreed‐upon manner to strike this balance, and the quantitative nature of this trade‐off is laboratory dependent. For screening tests that have an intrinsically poor sensitivity at low FF, a high failure rate may be advisable to prevent a high volume of FNs. Because the custom NIPS characterized here is sensitive and specific at low FF, it was possible to have a low test‐failure rate with few FNs and FPs (Figure 2b). Although there were two FNs at low FF, this number must be considered in context: 11‐fold more TPs were reported in this range (*n* = 22), and > 1800‐fold more women received confirmed or likely correct negative results (*n* = 3706). Had all low‐FF samples been failed in order to avoid the FNs, the result would have been no TNs, some ‘indirect positives’ attributable to the test failures that were indeed aneuploid (assuming every patient with a test failure got diagnostic testing) and approximately 3200 effective FPs (since guidelines recommend offering diagnostic follow‐up to patients with screen‐positive results or test failure^10^, ^13^). Importantly, the large number of diagnostic procedures associated with a high rate of test failure could have led to procedure‐related losses from invasive follow‐up. The clinical burden of managing thousands of test failures would be substantial and, as the utilization of NIPS expands in the average‐risk population^10^, ^13^, ^26^, minimizing this clinical burden will become increasingly important.

A low rate of test failure is important for reducing the clinical impact of NIPS (e.g. workflow burden and procedure‐related loss associated with follow‐up for test failures) but may also be important in minimizing the psychological impact on patients, which has yet to be investigated systematically. Uncertain results in diagnostic tests associated with pregnancy are already known to increase patient anxiety; for example, in a recent study of patients undergoing prenatal ultrasound examination because of abdominal pain and/or vaginal bleeding, uncertain results were associated with elevated anxiety levels, even higher than when a certain diagnosis of poor outcome was given^27^. Exploring the impact of uncertainty due to test failures and mitigating their occurrence are increasingly important as NIPS becomes a first‐line screening test in the general population.

In conclusion, our clinical experience in over 58 000 patients demonstrates that the customized WGS‐based NIPS approach without a FF threshold described in this study achieves high accuracy while also maintaining a low test‐failure rate of 0.1%. Use of a FF threshold would have impacted on thousands of patients, unnecessarily delaying the identification of a number of affected pregnancies. Failing samples based solely on FF is not necessary with a WGS‐approach that maintains high accuracy at low FF.

## Supporting information


**Appendix S1** Additional methods

**Figure S1** Outcome collection form.
**Figure S2** Distribution of maternal age (a), gestational age (b) and fetal fraction (c) in patients with (blue) and those without (red) reported pregnancy outcome. Traces indicate Gaussian kernel‐smoothed data for clarity. Vertical line in (a) shows advanced maternal‐age threshold of 35 years.

**Table S1** Robustness of ascertainment bias adjustment on inferred test performance
**Table S2** Evaluation of uncertainty of inferred test performance for low‐fetal‐fraction samples due to low sample size
**Table S3** Inferred test performance based on original algorithm used at time of patient testing
