# Supplementary material for: Clinical experience across the fetal‐fraction spectrum of a non‐invasive prenatal screening approach with low test‐failure rate
Source: Ultrasound Obstet Gynecol. 2020 Sep 1;56(3):422–30. doi: 10.1002/uog.21904 (PMC7496885; doi:10.1002/uog.21904)
Supplement: Supplementary file 1 — Appendix S1 Additional methods [file UOG-56-422-s001.docx]

**Appendix S1: Additional methods**

**Supplementary Text S1: Details of test-performance calculation adjustment**

Inferred sensitivity and inferred specificity calculations were adjusted for ascertainment bias using similar methodology to that described in Taneja *et al* [^1^](https://paperpile.com/c/NE3Whv/eefC). Here we show examples of the test-performance calculation adjustment for the full patient cohort reanalyzed with the updated algorithm when all aneuploidies are combined.

**Inferred sensitivity calculation example:**

Inferred sensitivity = TP/(TP +FN_sensitivity_adjusted_ )

An adjustment for FNs is needed to correct for differences in the rate of outcomes received for screen-positive patients compared to that for false negative outcomes. Of the 579 screen-positive patients identified, informative outcomes were returned for 244 (42.1%) patients. We assume that all false-negatives have been reported and therefore the FN response rate is 100% (the impact of this assumption is tested in Supplementary Text S2 and Supplementary Table S1).Response rate for screen-positives = # screen-positive outcomes/# screen-positive results # response rate for screen-positives.

FN_sensitivity_adjusted_= (FN_observed_) * (screen-positive response rate/false-negative self-reported rate) = 7*(42.1%/100%) = 2.95.

**Inferred specificity calculation example:**

Inferred specificity = TN/(TN +FP_specificity_adjusted_ )

An adjustment for FPs is needed to correct for differences in the rate of outcomes received for screen-negative patients compared to that for outcomes received for screen-positive patients. Response rate for screen-negatives = # screen-positive outcomes/# screen-negative results.

Of the 57,469 screen-negative patients identified, informative outcomes were obtained for 3,255 patients (5.7%).

FP_specificity_adjusted_= (FP_observed_) * (screen-negative response rate/screen-positive response rate) = 35*(5.7%/42.1%) = 4.7.

**Inferred negative predictive value (NPV) calculation example:**

Inferred NPV = TN/(TN + FN_NPV_adjusted_)

As above for inferred sensitivity, an adjustment for FNs is needed to correct for differences in the rate of outcomes received for screen-negative patients compared to that for false negatives.

FN_NPV_adjusted_ = (FN_observed_) * (screen-negative response rate/false-negative self-reported rate) = 7*5.7%= 0.4.

**Positive predictive value (PPV) calculation example:**

PPV = TP/(TP + FP)

Because we assume that TPs and FP outcomes are received at the same rate, we do not need an ascertainment bias correction for PPV. However, if these rates were different (as we assume in Supplementary Table S1), the ascertainment bias correction would be the relative rate of informative outcomes for TPs to the rate informative outcomes for FPs and the adjusted equation would be PPV = TP/(TP + FP_PPV_adjusted_) where:

FP_PPV_adjusted_ = (FP_observed_) * (TP response rate/FP response rate).

**Supplementary Text S2: Details of sensitivity analysis of test performance at low FF**

We used bootstrapping simulation[^2^](https://paperpile.com/c/NE3Whv/HNWj) to understand the impact of the relatively small sample size of positives at low FF on confidence intervals for inferred sensitivity and inferred specificity. For inferred sensitivity, we simulated TPs and FNs (TP_sim_ and FN_sim_, respectively) by drawing randomly from a size-matched cohort with the assumption that TP_sim_ occurred at the rate TP_observed_/(TP_observed_ + FN_observed_) and FN_sim_ occurred at the rate FN_observed_/(TP_observed_+FN_observed_). We then calculated the inferred sensitivity given the simulated number of TP_sim_ and FN_sim_, accounting for ascertainment bias as described in Supplementary Text S1. For inferred specificity, we followed a similar procedure. We simulated FP_sim_ and TN_sim_ assuming that the FP_sim_ occurred at the rate FP_observed_/(FP_observed_ + TN_observed_) and TN_sim_ occurred at the rate TN_observed_/(FP_observed_+TN_observed_). We then calculated the inferred specificity given the simulated number of FP_sim_ and TN_sim_, accounting for ascertainment bias. We repeated these simulations 10,000 times and report the 2.5% and 97.5% percentiles of these simulations in Supplementary Table S2. Supplementary Table S2 shows that uncertainty due to the smaller sample size at low FF yields similar confidence intervals of inferred sensitivity and inferred specificity as shown in Table 3 in the main manuscript.

**Pseudocode for bootstrapping simulation of inferred sensitivity and specificity confidence intervals:**

NUM_SIMS = 10000 # number of simulations

TP = *<number of true positives>*

TN = *<number of true negatives>*

FN = *<number of false negatives>*

FP = *<number of false positives>*

N_outcomes = TP + FN

N_pos_calls = TP + FP # screen-positive calls

TP_array = []

FN_array = []

TN_array = []

FP_array = []

sens_adj = []

spec_adj = []

p_FN = FN/(FN + TP)

p_FP = FP/(FP + TN)

function calc_FN_adj(TP_sim, TP_actual, FP, FN_sim, N_pos_calls):

N_pos_outcomes = TP_sim + FP

N_pos_calls_sims = N_pos_calls + (TP_sim - TP_actual)

N_frac_pos_outcomes = N_pos_outcomes/(N_pos_calls_sims)

return FN_sim * N_frac_pos_outcomes

function calc_FP_adj(TN_sim, TN_actual, FN, FP_sim, N_neg_calls):

N_neg_outcomes = TN_sim + FN

N_neg_calls_sims = N_neg_calls + (TN_sim - TN_actual)

N_frac_neg_outcomes = N_neg_outcomes/(N_neg_calls_sims)

return FP_adj = FP_sim * N_frac_neg_outcomes

for sim in NUM_SIMS:

# sensitivity

draws_sens = *<list of length N_outcomes of uniform random numbers from 0 to 1>*

FN_sim = len(draws_sens[draws_sens < p_FN]) # simulated number of FNs

TP_sim = len(draws_sens[draws_sens ≥ p_FN]) # simulated number of TPs

TP_array.append(TP_sim)

FN_adj = calc_FN_adj(TP_sim, TP, FP, FN_sim, N_pos_calls)

FN_array.append(FN_adj)

sens_adj.append(TP_sim/(TP_sim + FN_adj))

# specificity

draws_spec = *<list of length N_outcomes of uniform random numbers from 0 to 1>*

FP_sim = len(draws_spec[draws_spec < p_FP]) # simulated number of FNs

TN_sim = len(draws_spec[draws_spec ≥ p_FP]) # simulated number of TNs

TN_array.append(TN_sim)

FP_adj = calc_FP_adj(TN_sim, TN, FN, FP_sim, N_neg_calls)

FP_array.append(FP_adj)

spec_adj.append(TN_sim/(TN_sim + FP_adj))

lower_CI_sens = quantile(sens_adj, 0.025)

upper_CI_sens = quantile(sens_adj, 0.975)

lower_CI_spec = quantile(spec_adj, 0.025)

upper_CI_spec = quantile(spec_adj, 0.975)

save(sens_adj, spec_adj)

save(TP_array, FN_array, TN_array, FP_array)

**Supplementary Text S3: Details of simulated power analysis for determining differences in inferred sensitivity at low and high FF**

We used a simulation approach to estimate the power to detect differences in the detection rate at FF levels above and below 4%.[^3^](https://paperpile.com/c/NE3Whv/wCr9) Using the bootstrapping procedure described in Supplementary Text S2, we simulated inferred sensitivity (detection rate) at FF levels above and below 4% 10,000 times, and we calculated the Fisher’s exact test to assess significance of differences in inferred sensitivity between FF levels above and below 4% for each simulation. We then calculated the percentage of simulations that resulted in a significant p-value (i.e., p<0.05). When all aneuploidies were combined, 2.52% of simulations resulted in a significant p-value, suggesting that the aneuploidy detection rate is not significantly different above and below 4% FF. For trisomy 21, 1.49% of simulations resulted in a significant p-value, suggesting that the detection rate for trisomy 21 is not significantly different above and below 4% FF. For trisomy 18 and trisomy 13, no significantly different p-values were observed due to few samples at low FF.

To determine the inferred sensitivity at low FF needed to reach a power of 80%, we built on the analysis described above, repeating the analysis with a different number of respective true positives and false negatives in the low FF patients, while maintaining the overall number of true positives and false negatives in the dataset. We then determined the highest inferred sensitivity value at low FF for which at 80% of simulations resulted in a significant p-value.

**Pseudocode for power analysis simulation:**

*<Compute analysis detailed in Supplementary Text S2 for patients with FF ≥4% and read in TP_array and FN_array as TP_array_high_FF and FN_array_high_FF, respectively>*

TP_actual_low_FF = *<number of low-FF TPs from empirical results>*

FN_actual_low_FF = *<number of low-FF FNs from empirical results>*

FP_actual_low_FF = *<number of low-FF FPs from empirical results>*

sens_underlying = []

NUM_SIMS = 10000 # number of simulations

# vary the number of underlying TPs and FNs at low FF

for TP_i in range(0:TP_actual_low_FF):

FN_i = FN_actual_low_FF + TP_actual_low_FF - TP_i

*<Compute analysis detailed in Supplementary Text S2 for patients with FF < 4% saving TP_array and FN_array as TP_array_i and FN_array_i, respectively>*

N_pos_calls = TP_i + FP_actual_low_FF

FN_i_adj = calc_FN_adj(TP_i, TP_actual_low_FF, FP_actual_low_FF, FN_i, N_pos_calls)

sens_underlying.append(TP_i/(TP_i + FN_i_adj))

p_value_array = []

for j in range(NUM_SIMS)):

p_value_array.append(*<Fisher’s exact test comparing inferred sensitivity for patients with FF < 4% (TP_array_i[j] and FN_array_i[j]) and inferred sensitivity for patients with FF ≥4% (TP_array_high_FF[j] and FN_array_high_FF[j])>*)

percent_significant_p_values = sum(p_value_array < 0.05) / NUM_SIMS

*<find index i in percent_significant_p_values where value drops below 80%>*

return sens_underlying[i-1]

**Supplementary Text S4: Comparison of low FF and high FF false negative rates**

As an alternative methodology to determine differences in sensitivity between low FF and high FF (using the reanalyzed data, Table 2) without needing to account for ascertainment bias, we quantified the ratio of false negatives rates of low FF to that for high FF, akin to a relative risk. The 95% confidence interval of this ratio was determined assuming the log of the false-negatives rates is approximately normally distributed. The ratio of false-negative rates for all trisomies combined was 3.20, 95% confidence intervals: (0.66, 15.60). Because the confidence interval spans 1, there is insufficient evidence to conclude that the false-negative rates of low-FF and high-FF are significantly different.

References

1. [Taneja PA, Snyder HL, de Feo E, Kruglyak KM, Halks-Miller M, Curnow KJ, Bhatt S. Noninvasive prenatal testing in the general obstetric population: clinical performance and counseling considerations in over 85 000 cases. *Prenat Diagn*. 2016;36(3):237-243.](http://paperpile.com/b/NE3Whv/eefC)

2. [Efron B. Bootstrap Methods: Another Look at the Jackknife. In: Kotz S, Johnson NL, eds. *Breakthroughs in Statistics: Methodology and Distribution*. New York, NY: Springer New York; 1992:569-593.](http://paperpile.com/b/NE3Whv/HNWj)

3. [Arnold BF, Hogan DR, Colford JM Jr, Hubbard AE. Simulation methods to estimate design power: an overview for applied research. *BMC Med Res Methodol*. 2011;11:94.](http://paperpile.com/b/NE3Whv/wCr9)
