# Supplementary material for: Clinical experience across the fetal‐fraction spectrum of a non‐invasive prenatal screening approach with low test‐failure rate
Source: Ultrasound Obstet Gynecol. 2020 Sep 1;56(3):422–30. doi: 10.1002/uog.21904 (PMC7496885; doi:10.1002/uog.21904)

**Supplementary figures**

**Figure S1: Outcome collection form**

**
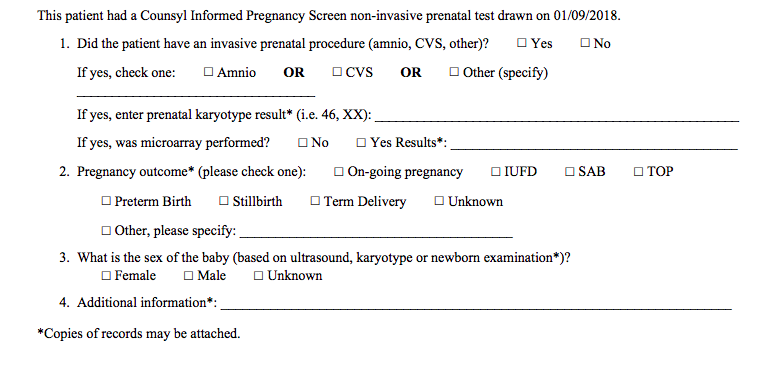
**

**Figure S2:** Distribution of maternal age (a), gestational age (b) and fetal fraction (c) in patients with (blue) and those without (red) reported pregnancy outcome. Traces indicate gaussian kernel-smoothed data for clarity. Vertical line in (a) shows advanced maternal age threshold of 35 years.


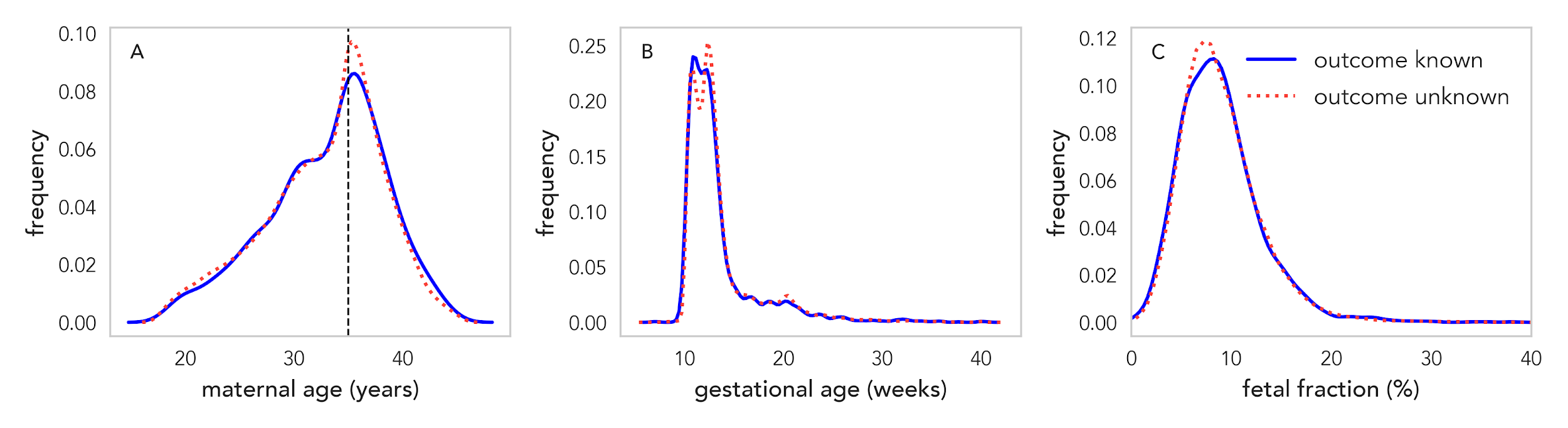

Supplement: Supplementary file 2 — Figure S1 Outcome collection form. Figure S2 Distribution of maternal age (a), gestational age (b) and fetal fraction (c) in patients with (blue) and those without (red) reported pregnancy outcome. Traces indicate Gaussian kernel‐smoothed data for clarity. Vertical line in (a) shows advanced maternal‐age threshold of 35 years. [file UOG-56-422-s002.docx]
