# Supplementary material for: Clinical experience across the fetal‐fraction spectrum of a non‐invasive prenatal screening approach with low test‐failure rate
Source: Ultrasound Obstet Gynecol. 2020 Sep 1;56(3):422–30. doi: 10.1002/uog.21904 (PMC7496885; doi:10.1002/uog.21904)
Supplement: Supplementary file 3 — Table S1 Robustness of ascertainment bias adjustment on inferred test performance Table S2 Evaluation of uncertainty of inferred test performance for low‐fetal‐fraction samples due to low sample size Table S3 Inferred test performance based on original algorithm used at time of patient testing [file UOG-56-422-s003.doc]

**Supplementary Tables**

**Table S1: Robustness of ascertainment bias adjustment on inferred test performance.**

*Overall test performance combining all three aneuploidies (Table 3) was reanalyzed assuming different outcome response rates for FNs and FPs. Inferred test performance metrics were then recalculated based on outcome response rate assumptions and resulting ascertainment bias adjustments, as detailed in Methods and Supplementary Text S1. Results show that inferred test performance is robust to wide variation in outcome response rates.*

|  | **Base** | **Minimum** | **Maximum** | **Base** | **Minimum** | **Maximum** |
| --- | --- | --- | --- | --- | --- | --- |
|  | **Response rate** | **Response rate** | **Response rate** | **performance** | **performance** | **performance** |
| **INFERRED** | 100% | 60% | 100% | 98.60% | 97.70% | 98.60% |
| **SENSITIVITY:** |  |  |  |  |  |  |
| Adjust FN based on response rate |  |  |  |  |  |  |
| **INFERRED** | 42% | 10% | 70% | 99.86% | 99.39% | 99.91% |
| **SPECIFICITY:** |  |  |  |  |  |  |
| Adjust FP based on response rate |  |  |  |  |  |  |
| **INFERRED** | 100% | 60% | 100% | 85.70% | 78.20% | 85.70% |
| **PPV:** |  |  |  |  |  |  |
| Adjust FP based on response rate |  |  |  |  |  |  |
| **INFERRED** | 100% | 60% | 100% | 99.99% | 99.98% | 99.99% |
| **NPV:** |  |  |  |  |  |  |
| Adjust FN based on response rate |  |  |  |  |  |  |

**Table S2: Evaluation of uncertainty of inferred test performance for low-FF samples due to low sample size.**

***Simulated confidence intervals for low-FF inferred sensitivity and inferred specificity due to the relatively small sample size of positives at low FF, as described in Supplementary Text S2. The confidence intervals of inferred sensitivity and inferred specificity at low FF are comparable to the uncertainty ranges reported in Table 3. (* because no false-negatives were reported for T13, the simulations were not applicable; see Supplementary Text S2.)***

|  | **Trisomy 21** | **Trisomy 18** | **Trisomy 13** | **Total** |
| --- | --- | --- | --- | --- |
| **INFERRED** | (86.2%, 100.0%) | (84.0%, 100.0%) | (n/a, n/a)* | (88.9%, 100.0%) |
| **SENSITIVITY** |
| **INFERRED** | (99.5%, 100.0%) | (99.7%, 100.0%) | (99.6%, 100.0%) | (99.1%, 99.8%) |
| **SPECIFICITY** |

*Cannot compute confidence intervals because of small sample size

**Table S3: Inferred test performance based on original algorithm used at time of patient testing.**

|  | **Inferred Sensitivity** | | | | **Inferred Specificity** | | | |
| --- | --- | --- | --- | --- | --- | --- | --- | --- |
|  | **Trisomy 21** | **Trisomy 18** | **Trisomy 13** | **Total** | **Trisomy 21** | **Trisomy 18** | **Trisomy 13** | **Total** |
| **All patients** | *99.1%*  *(98.0%, 99.2%)* | *96.0%*  *(90.0%, 96.4%)* | *94.3%*  *(88.2%, 96.3%)* | *98.0%*  *(95.4%, 98.2%)* | *99.97%*  *(99.63%, 99.99%)* | *99.96%*  *(99.83%, 99.99%)* | *99.94%*  *(99.91%, 99.97%)* | *99.87%*  *(99.37%, 99.95%)* |
| **FF < 4%** | *87.9%*  *(76.9%, 88.5%)* | *89.5%*  *(77.8%, 90.9%)* | *100.0%*  *(n/a, n/a)** | *89.7%*  *(79.2%, 91.1%)* | *99.94%*  *(99.62%, 99.97%)* | *99.87%*  *(99.60%, 99.95%)* | *99.82%*  *(99.76%, 99.92%)* | *99.63%*  *(98.97%, 99.84%)* |
| **FF >= 4%** | *100.0%*  *(100.0%, 100.0%)* | *97.2%*  *(92.7%, 97.4%)* | *93.4%*  *(86.7%, 95.7%)* | *98.9%*  *(97.4%, 99.0%)* | *99.98%*  *(99.63%, 99.99%)* | *99.97%*  *(99.85%, 99.99%)* | *99.95%*  *(99.92%, 99.98%)* | *99.89%*  *(99.39%, 99.95%)* |
|  | **PPV** | | | | **Inferred NPV** | | | |
|  | **Trisomy 21** | **Trisomy 18** | **Trisomy 13** | **Total** | **Trisomy 21** | **Trisomy 18** | **Trisomy 13** | **Total** |
| **All patients** | *95.4%*  *(92.1%, 98.7%)* | *84.9%*  *(75.3%, 94.5%)* | *48.4%*  *(30.8%, 66.0%)* | *86.9%*  *(82.6%, 91.2%)* | *99.99%*  *(99.97%, 100.00%)* | *99.99%*  *(99.96%, 100.00%)* | *>99.99%*  *(99.98%, 100.00%)* | *99.98%*  *(99.94%, 100.00%)* |
| **FF < 4%** | *90.9%*  *(73.9%, 100.0%)* | *77.8%*  *(50.6%, 100.0%)* | *40.0%*  *(0.0%, 82.9%)* | *76.0%*  *(59.3%, 92.7%)* | *99.92%*  *(99.56%, 100.00%)* | *99.95%*  *(99.66%, 100.00%)* | *100.00%*  *(n/a, n/a)** | *99.86%*  *(99.39%, 100.00%)* |
| **FF >= 4%** | *95.8%*  *(92.5%, 99.1%)* | *86.4%*  *(76.2%, 96.5%)* | *50.0%*  *(30.8%, 69.2%)* | *88.2%*  *(83.9%, 92.5%)* | *100.00%*  *(n/a, n/a)** | *99.99%*  *(99.97%, 100.00%)* | *>99.99%*  *(99.98%, 100.00%)* | *99.99%*  *(99.96%, 100.00%)* |

Test performance results are based on results at the time of patient screening. Calculations and error boundaries (shown in parentheses) are described in Methods. *Cannot compute confidence because of small sample size.
